# Leaky Gut: Effect of Dietary Fiber and Fats on Microbiome and Intestinal Barrier

**DOI:** 10.3390/ijms22147613

**Published:** 2021-07-16

**Authors:** Haruki Usuda, Takayuki Okamoto, Koichiro Wada

**Affiliations:** Department of Pharmacology, Faculty of Medicine, Shimane University, 89-1 Enyachō, Izumo 693-8501, Japan; koiwada@med.shimane-u.ac.jp

**Keywords:** leaky gut, intestinal permeability, short-chain fatty acids, tight junction, microbiome

## Abstract

Intestinal tract is the boundary that prevents harmful molecules from invading into the mucosal tissue, followed by systemic circulation. Intestinal permeability is an index for intestinal barrier integrity. Intestinal permeability has been shown to increase in various diseases—not only intestinal inflammatory diseases, but also systemic diseases, including diabetes, chronic kidney dysfunction, cancer, and cardiovascular diseases. Chronic increase of intestinal permeability is termed ‘leaky gut’ which is observed in the patients and animal models of these diseases. This state often correlates with the disease state. In addition, recent studies have revealed that gut microbiota affects intestinal and systemic heath conditions via their metabolite, especially short-chain fatty acids and lipopolysaccharides, which can trigger leaky gut. The etiology of leaky gut is still unknown; however, recent studies have uncovered exogenous factors that can modulate intestinal permeability. Nutrients are closely related to intestinal health and permeability that are actively investigated as a hot topic of scientific research. Here, we will review the effect of nutrients on intestinal permeability and microbiome for a better understanding of leaky gut and a possible mechanism of increase in intestinal permeability.

## 1. Introduction

Leaky gut refers to the dysfunction of the intestinal barrier and often leads to the generation of leaky gut syndrome (LGS) under chronic states. As the name indicates, the pathological manifestation of leaky gut is increased intestinal permeability, which is induced by various causative factors. The term ‘intestinal permeability’ is simple; however, the definition of intestinal permeability is ambiguous, since it potentially has two meanings—epithelium permeability and permeability of capillary vessels in villi. It is difficult to evaluate the latter in vivo. So, the term ‘leaky gut’ is used to indicate abnormal translocation of big-size molecules from lumen to villi or excessive absorption of such molecules from lumen into systemic circulation, which, in turn, induces various organ disorders. Historically, the concept of leaky gut began to emerge as epithelium permeability. Classical LGS has been observed concomitantly with gut inflammation, including inflammatory bowel diseases (IBD) or coeliac disease, and NSAIDs-induced ulceration that was studied from 1970 to 1990 extensively [1,2,3]. Hence, leaky gut was recognized as a typical manifestation of gut inflammation during this period and ceased to be focused on. Leaky gut has again come under the scientific spotlight due to the attention gained by the gut microbiome. Genome-sequencing methods have dramatically evolved within the last two decades, which now enables analysis of the whole intestinal bacterial genome, or identification of bacteria in stool sample by sequencing conserved marker genes such as the 16srRNA gene of bacteria [4]. Now we know the entire genetic content of human gut microbiota, the amount of which is calculated to be 100-fold or higher compared to human genomic content [5]. These findings have led to the emergence of the concept of intestinal enterotypes. Two major phyla, *Bacteroidetes* and *Firmicutes*, dominate among the microbiota of humans, and many studies have shown the considerable inter- and intrapersonal variability at the genus level and above [6]. *Firmicutes* phylum contains various types of bacteria including facultative, anaerobic, cocci, and bacilli bacteria. Gram-positive bacteria are major in this phylum and have relatively low guanine and cytosine contents [7,8]. On the contrary to *Firmicutes*, gram-negative bacteria are dominant in *Bacteroidetes* phylum and human gut have *Bacteroides*, *Alistipes*, *Parabacteroides*, and *Prevotella* genus primarily [9]. Bacteria interacts with a host directly or indirectly via physiological active molecules secreted from bacteria including short-chain fatty acids (SCFAs), p-cresol, p-cresyl-glucuronide (pCG), indoxyl sulphate (IS), indole-3 acetic acid (IAA), and H_2_S and trimethylamine N-oxide (TMAO) that may result in protective or harmful effects on the intestinal barrier and various organs distant from the intestine including the liver, kidney, and brain [10,11]. In fact, dysbiosis, an unhealthy alternation of gut microbiota, correlates with several types of diseases, including IBD [12], cancer [13], neuropsychiatric disorders [14], chronic kidney diseases (CKD) [15,16], and cardiometabolic diseases including obesity, type 2 diabetes (T2D), and cardiovascular disease [17], along with increased intestinal permeability. Dysbiosis results in an increased population of pathogenic bacteria which are likely to produce higher levels of lipopolysaccharides (LPS) and induce damage to epithelial cells which is one of the possible mechanisms of increased intestinal permeability observed with dysbiosis [18,19,20]. Furthermore, disruptions of intercellular connections let harmful molecules, including LPS, invade the intestinal tissue, and consequently access the blood stream, which provokes or worsens not only IBD [21] but also systemic diseases [18,19,20]. Hence, the concept of LGS shifted from a mere inflammatory phenotype to an exacerbation factor of systemic diseases, although its etiology is still unclear. In addition, bidirectional translocation of molecules, metabolites, and toxins derived from systemic circulation to gut lumen also can be recognized as LGS although the evidence is poor.

**Figure 1:**
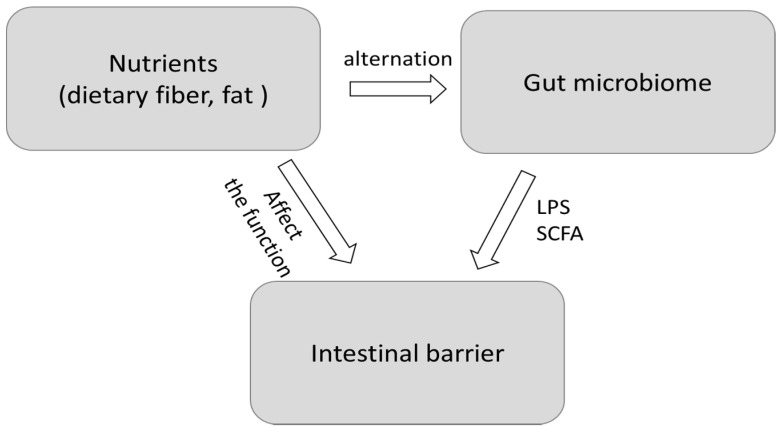
Relationships between nutrients, gut microbiome, and intestinal barrier. LPS: lipopolysaccharides, SCFAs: short-chain fatty acids.

Dietary components come into contact with intestinal lumen for a long time and are likely to regulate gut microbiota and intestinal permeability. However, many studies which are now focusing on gut microbiota recognize alteration of intestinal permeability as an ancillary symptom. In this review, we provide an overview of recent studies that investigate the effect of dietary components on intestinal permeability and microbiome and hypothesize a possible correlation among them (Figure 1). Especially, the effects of dietary fiber and a high fat diet (HFD) on microbiome and intestinal permeability have been attracting attention globally as targets of research and are investigated most actively. Hence, we will focus on these nutrients.

## 2. Intestinal Barrier

Luminal components including unstirred water, glycocalyx, and mucus as well as antibacterial molecules including defensins, lysosome, and IgA provide the first line of defense before harmful bacteria come in contact with the epithelium. In addition, microclimate and secretions from the stomach, pancreas, and pancreatic acids destroy bacteria and antigens in the lumen [22].

Mucus layers and epithelium are the most important and major structures of intestinal barrier. A mucus layer exists on the outer surface of the large intestine. This layer is composed of two sub-layers. The outer layer is thick and loose, where bacteria and bacteria-derived molecules are abundant. Many species of commensal bacteria grow and form colonies on the outer layer, such that under healthy conditions, pathogenic bacteria cannot overgrow or invade this territory of commensal bacteria. The inner layer is firm, adherent, and harbors quite small numbers of bacteria. This layer acts as a boundary between bacteria and epithelium [23]. In the case of the small intestine, the mucus layer is single and fluid and contains abundant antimicrobial substances. Studies using experimental animals have shown that the disruption of mucus production can lead to intestinal damage and inflammation [24].

**Figure 2:**
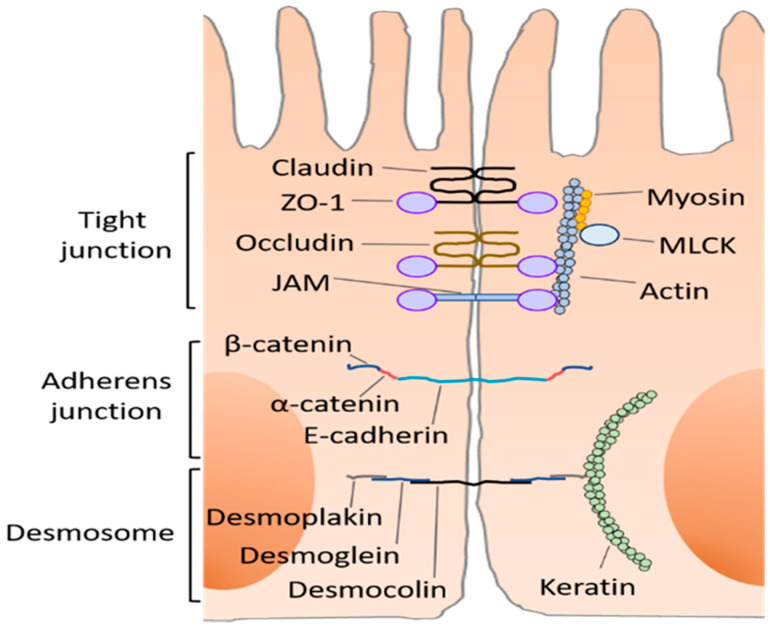
Construction of intestinal barrier.

The epithelium is located just below inner layers and is composed of normal epithelial cells and several types of cells possessing specific functions, including Paneth cells, goblet cells, etc. Paneth cells secrete antibacterial peptides such as lysozyme and defensins and prevent colonization of harmful bacteria [25], while enterocytes produce chloride in response to noxious stimuli [26]. Goblet cells contribute to maintaining a mucosal layer by secreting mucin [27]. Epithelial cells are linked by the apical junctional complex which consists of the tight junction (TJ) and adherence junction. TJ is further composed of claudin, occludin, and junctional adhesion protein molecule-A (JAM-A) as well as intracellular plaque proteins such as zonula occludens (ZOs) and cingulin [28] (Figure 2). Twenty-seven human claudin genes have been identified, though the protein expression of some of them is not confirmed [29]. Of note, claudin-13 is expressed in rodents but is absent in humans [30]. Claudin and occludin function cooperatively. Nevertheless, embryonic stem cells that lack occludin differentiate into polarized epithelia with functional TJ [31]. In addition, occludin knockout leads to the normal barrier function of intestinal epithelium [32]. Thus, occluding plays a supportive role in intestinal permeability.

**Figure 3:**
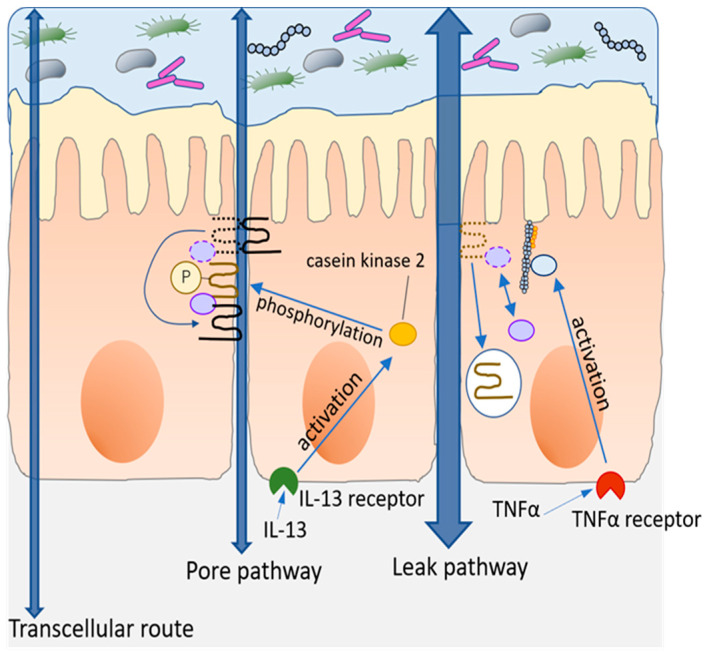
The mode of action for opening pore and leak pathways.

Molecules can pass through the epithelium passively via transcellular route [26,33] or paracellular route [26,34] (Figure 3). Soluble lipids, small hydrophilic compounds, ions, and water molecules pass through the transcellular route. The paracellular route allows the passage of bigger molecules, although the size is limited at up to 600 Da in in vivo and 10 kDa in in vitro [35,36] via apical junctional complex. The latter route is further divided into two types of pathways and regulated by IL-13 and tumor necrosis factor (TNF). IL-13 specifically increases flux across the small molecules, including ions and water, through the pore pathway [37]. IL-13 causes barrier loss by inducing claudin-2 expression as well as by increasing apoptosis and inhibiting wound healing both in vitro and in vivo, whereas lower doses of IL-13 induce claudin-2 upregulation and claudin-2-dependent pore-pathway activation in response to IL-13 exposure without increase in leaks or unrestricted pathway flux [37,38]. TNF opens the leak pathway via myosin light chain kinase (MLCK) and lets bigger molecules pass through the intracellular space [37]. Hence, the leak pathway is likely to be associated with inflammation, which would permit the passage of macromolecules, bacterial products, and food antigens. TNFα has also been shown to regulate TJ and the clinically relevant role of TNF in IBD pathogenesis is clearly demonstrated by the efficacy of anti-TNF antibodies in IBD, which reduces disease severity and restores intestinal barrier function [39]. Restoration of epithelial barrier function by anti-TNF therapy may reflect mucosal healing in the setting of a dampened immune system; however, pre-clinical studies have shown that TNF signaling also modulates TJ. Many studies have shown decreased levels of intestinal TJ-consisting proteins in animal models of IBD [40], obesity, and T2D [41,42], with increased permeability, suggesting that a leaky pathway is responsible for LGS.

Epithelial cells bind each other with tight junction, adherens junction, and desmosome. Tight junction consists of claudin, occluding, and junctional adhesion protein molecule-A (JAM) recognized as a key construction which regulate the absorption of molecules via the paracellular route. These molecules, associated with zonula occluden-1 (ZO-1), which contribute to the formation of tight junction as a scaffold protein. Myosin-actin complex which is associated with myosin light-chain kinase (MLCK) is also interacting with ZO-1 in a steady state. Adherens junction is comprised by *α*-catenin, *β*-catenin, and E-cadherin. Desmosome consists of three kinds of molecules with binding to keratin.

Molecules are absorbed from intestinal lumen into the tissue via three pathways. Lipid soluble molecules, small hydrophilic molecules, ions, and water pass through the cell body. Ions and water are also absorbed intracellularly which is called the pore pathway. Bigger molecules (>600 Da) can be absorbed when the tight junction becomes loose or collapses by inflammation or other harmful stimuli. The modes of action for opening pore pathway by interleukin-13 (IL-13) and leak pathway by tumor necrosis factor (TNF-*α*) are relatively well known. Signaling from IL-13 receptor, activate casein kinase 2 and phosphorylate occluding successively which allows the interaction of claudin-2 and occluding linking with zonula occluden-1 (ZO-1) as shown in Figure 3. In the case of TNF-*α*, myosin light-chain kinase (MLCK) is activated and endocytosis of occluding is promoted which results in the collapse of tight junction.

## 3. How to Evaluate Intestinal Barrier Function

Oral administration of evaluating-reagents is a standard method and carried out most frequently in in vivo study. Especially, the administration of two non-metabolized sugars, lactulose and mannitol, is used as a golden standard method for human-targeted studies [43]. The absorption of lactulose is increased when the paracellular epithelial barrier is compromised whereas the smaller sized mannitol is constantly absorbed regardless of barrier function, which reflects the basal ability of intestinal absorption [43]. Hence, the value of urine lactulose/mannitol ratio is used as an index of disruption of the intestinal barrier [43]. However, these sugars can detect the status of barrier function in the small intestine only since these are degraded by bacteria [43]. Sucralose and ^51^Cr-ethylenediaminetetraacetic acid (EDTA) can evaluate whole intestinal permeability since these molecules are not metabolized by bacteria and used widely from in vitro studies to clinical studies [44].

The size of these sugars and ^51^Cr-(EDTA is small so that more big-sized molecules which are not non-metabolized by human enzymes and bacteria are also used in animal studies, in vitro and ex vivo, to evaluate absorption of molecules from the leak pathway. Ovalbumin (OVA) is frequently used to measure antigen uptake following oral gavage [43]. Polyethylene glycol and fluorescein isothiocyanate (FITC)-dextran are convenient to estimate the size of the molecule that leaks from the lumen since various molecular sizes of them are available. The molecular weight of these reagents which are most frequently used is around 4000 [45].

There are several other methods to evaluate permeability. Calculating the electrical resistance of the cell membrane, which reflects paracellular ion permeability, is specifically applicable to in vitro and ex vivo study [46]. In addition, various biomarkers relating to epithelial damage including citrulline, fatty acid-binding proteins (FABP) and LPS have been used as an indirect index of decreased intestinal barrier as shown elsewhere in detail [45].

## 4. Dietary Fiber

### 4.1. Dietary Fiber and Intestinal Barrier

#### 4.1.1. Fermentation of Dietary Fiber and SCFA

Dietary fibers are largely metabolized by gut bacteria. This is because they can break down various types of carbohydrates since they encode over 260 glycoside hydrolases for the degradation of carbohydrates whereas a human has only 17 enzymes for digestion [47], meaning that human themselves are poor at digesting a variety of dietary fiber. In addition, more than 100 trillion bacteria can be engaged in consuming carbohydrates derived from dietary fiber [39] that would help effective digestion of dietary fiber. Hence, dietary fibers resistant to digestion by the host are termed as microbiota-accessible carbohydrates (MACs) [48]. Dietary fibers are divided into soluble and insoluble fibers. Insoluble fiber includes cellulose, some hemicellulose, and lignin [49]. Soluble fiber encompasses wheat dextrin, pectin, gums, β-glucan, psyllium, and fructans, as well as some hemicellulose [49]. These fibers are derived from grains, fruits, vegetables, and legumes [50]. Generally, insoluble fibers are poorly fermented by gut microbes, but they likely promote the gut transit rate and thus reduce the amount of time available for colonic bacterial fermentation of non-digested foodstuff [51]. Soluble fibers can be further processed by bacteria into SCFAs as metabolites [52] although some of them are not fermentable including psyllium and gums.

Different types of bacteria produce different types of SCFAs [53,54,55,56,57]. The most abundant SCFAs in the human colon are acetate, propionate, and butyrate, with a molar ratio of 60:20:20, approximately [58]. Colonocytes absorb SCFAs via transporters or simple diffusion. SCFA are transported to various organs via solute carrier family 16 member 1 (SLC16a1) and SLC5a8, transporters for SCFAs [59]. A majority of the acetate bypasses the splanchnic circulation to be converted into acetyl-CoA for lipogenesis or oxidation in peripheral muscles. The remaining acetate is converted to butyrate and is used by colonocytes [60,61]. Propionate primarily contributes to gluconeogenesis in liver. Some bacteria species intake lactate and succinate and convert them into propionates. These physiological functions are regulated by SCFAs via G-protein coupled receptors including GPR40, GPR41, GPR43, and GPR120, which are distributed across various types of cells. GPR41 and GPR43 are highly expressed in the intestine [62]. It has been reported that, in humans, the fecal SCFA level increases after intaking an MACs-rich diet [63].

#### 4.1.2. Contribution of SCFA to Intestinal Barrier

**Figure 4:**
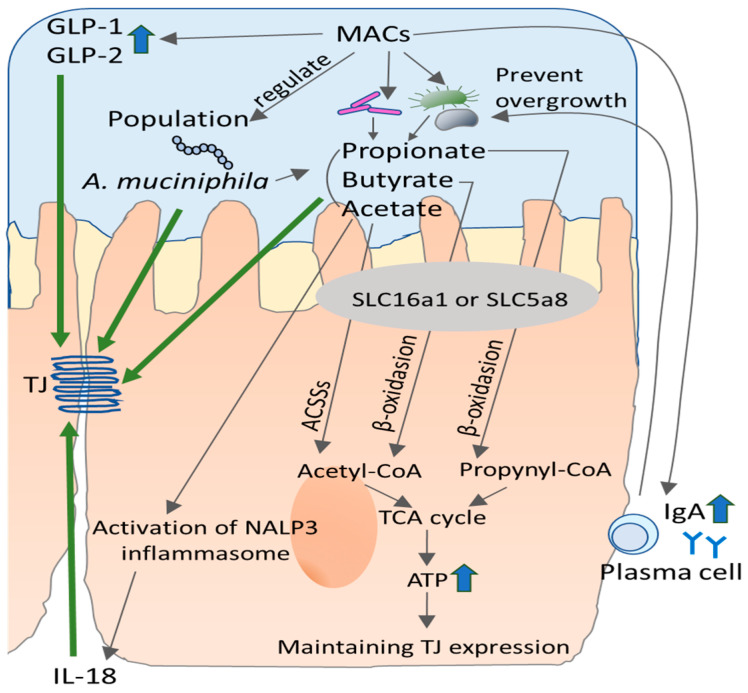
Effects of microbiota-accessible carbohydrates (MACs) on tight junction (TJ). Intestinal bacteria digest fermentable MACs and produce short-chain fatty acids (SFCA) which are absorbed into epithelial cells via solute carrier family (SLC) transporter or simple diffusion. Acetate, butyrate, and propionate are converted to acetyl-CoA or propynyl-CoA via acetyl-CoA carboxylases (ACSSs) or *β*-oxidation to produce ATP which maintain cell homeostasis including the function of TJ. Acetate activates nucleotide-binding oligomerization domain 3 (NLRP3) and promotes the secretion of IL-18 from epithelial cells, which contributes to TJ function. Other SCFA also have a protective effect on TJ. MACs also may contribute to TJ function via regulating the growth of *Akkermansia muciniphilla* (*A. muciniphilla*) or the production of Glucagon-Like Peptide (GLP) 1 and GLP-2.

Butyrate is a well-documented beneficial factor for maintaining colonocyte health by providing energy to intestinal epithelial cells, which likely contributes to intestinal epithelial integrity [64]. Butyrate suppresses cytokine-induced barrier dysfunction by modifying claudin-2 levels in vitro [65]. Animal studies have also shown similar protective effects on not only the intestinal barrier [66,67] but also the airway epithelial barrier [68]. Fecal calprotectin, a marker of intestinal inflammation, is diminished by administration of butyrate to UC patients [69]. In addition to these effects, butyrate seems to maintain intestinal integrity by inducing colonic mucin expression as shown in an in vitro study [70]. Besides, animal studies show that acetate directly activates nucleotide-binding oligomerization domain 3 (NLRP3) inflammasome in gut epithelial cells which results in the release of IL-18 [71], which, in turn, promotes intestinal barrier integrity via activation of IL-18 receptor on the mice epithelial cells [72]. However, genetical knocking out of IL-18 in the whole body or blocking of IL-18 receptor enhances the sensitivity to chemicals which induces colitis in mice [73,74], suggesting that acetate contributes to maintaining the epithelial barrier via NLRP3 signaling, followed by IL-18 production. Propionate also diminishes hyper intestinal permeability induced by dextran sulfate sodium (DSS) via amelioration of downregulation of ZO-1, occludin, and E-cadherin expressions in the colonic tissue in mice [75]. Recently, acetate, propionate, and butyrate have been shown to synergistically promote intracellular permeability by modifying TJ expression or distribution, including ZO-1 [76] in vitro. Together with these findings, SFCAs are recognized as a key factor for maintaining the intestinal barrier (Figure 4).

#### 4.1.3. Contribution of Dietary Fiber to Intestinal Barrier Integrity

The impact of MACs on intestinal permeability has been investigated through two kinds of approaches using a fiber-deprived or high fiber diet. Mice fed on an MACs-deprived diet exhibited severe colitis and increased intestinal permeability induced by DSS along with less serum IL-18 levels in mice [71]. A recent animal study targeting mice demonstrated that the removal of MACs affects the secretions of glucagon-like peptide-1 (GLP-1) and GLP-2, which synergistically ameliorate intestinal injury and improve intestinal healing [77,78]: they are downregulated in ileum and colon, along with increased intestinal permeability [79]. These results indicate that MACs contribute to the intestinal barrier via regulating the intestinal immune system and regulation of secretion of peptide hormones from colonocytes (Figure 4). Besides, the supplementation of fructo-oligosaccharides that are produced by degradation of inulin, a representative water-soluble dietary fiber, induces IgA production in rat cecum and suppresses a MACs-deficient diet-induced decrease in intestinal permeability [80]. In addition, the severity of DSS-induced colitis is diminished after administration of an MACs-containing diet, along with a marked increase in fecal butyrate [81]. These results also suggest that MACs have beneficial effects on the intestinal barrier. Nevertheless, whether MAC supplementation is really recommended to maintain intestinal health including barrier function is not confirmed yet since some clinical studies show beneficial effects of fructo-oligosaccharides and polydextrose on intestinal barrier function in healthy subjects or patients of pancreatitis [82,83], but administration of oat β-glucan, arabinoxylan (soluble hemicellulose) do not show significant effects on acute indomethacin-induced intestinal hyperpermeability [84,85]. Oligofructose-enriched inulin also does not improve intestinal barrier function effective in the patients of celiac disease [84,85].

### 4.2. MACs-Induced Alteration of Microbiota and Intestinal Barrier Integrity

Several studies have shown alteration of microbiota after MAC administration. The fecal microbiota composition of HFD-fed mice is modified by inulin administration in a dose-dependent manner. In this study, populations of *Roseburia, Clostridium* I, IV, and XIV spp. decreased, whereas the levels of *Bifidobacterium* spp. and *Bacteroidetes* increased [86], along with decreased caloric intake. Another study targeting humans with mild constipation also demonstrated that inulin induces an increase in the populations of *Anaerostipes*, *Bilophila*, and *Bifidobacterium* genus [87]. Especially, *Bilophila* spp. have been reported to be associated with softer stool and a favorable change in constipation-specific quality-of-life measures [87]. In addition, T2D is associated with reduced abundance of fiber-degrading bacteria in humans [88,89]. On the other hand, prolonged low-fiber feeding aggravated allergic airway disease in mice, which could be corrected by administration of the SCFA propionate [90], implying alternation of microbiota. These results suggest that MACs exhibit a favorable effect on microbiota and intestinal health that would affect the state of various systemic diseases, although the precise mechanism remains unknown. Besides, an MAC-deprived diet leads to an increase in levels of *Bacteroides thetaiotaomicron* (*B. thetaiotaomicron*), which intakes intestinal mucus glycans in mice [48]. Of note, microbiota transplant to germ-free mice showed that the population of mucin-degrading bacteria including *B. thetaiotaomicron* and *Akkermansia muciniphila* (*A. muciniphila*) increased in the intestine under deficiency of dietary MACs [91,92]. Interestingly, animal study shows that *A. muciniphila* is a promising probiotic bacteria despite its mucin-degrading feature [93]. *A. muciniphila* accounts for 1–4% of human gut microbiota starting from early life [94]. Decreased population of *A. muciniphila* has been reported in patients with IBD [95,96]. Conversely, the population of intestinal *A. muciniphila* increased in accordance with DSS-induced colitis in mice [97,98]. However, intervention studies indicate protective effects of *A. muciniphila* on intestinal integrity or inflammatory states in an animal colitis model. Administration of live *A. muciniphila* ameliorates DSS-induced colitis along with suppression of increase in intestinal permeability in mice [99]. Another animal study demonstrated that spleen weight, colon inflammation index, and colon histological score as well as the expression of the pro-inflammatory cytokines including TNF-α and IFN-γ in the colon are decreased by the administration of *A. muciniphila* [100]. Furthermore, outer membrane protein derived from *A. muciniphila* also shows similar protective effects in a mice colitis model [101]. This protein has been shown to enhance intracellular permeability, activate signaling pathway through Toll-like receptor 2 (TLR2) and TLR4, and modulate cytokine production from peripheral blood mononuclear cells in vitro [102]. The mode of action of *A. muciniphila* in serving these effects is still unclear. Further investigation might disclose this mechanism in the future.

## 5. HFD and Bile Acids

### 5.1. HFD and Intestinal Barrier

#### 5.1.1. Effects of Fatty Acid and HFD on Intestinal Barrier

**Figure 5:**
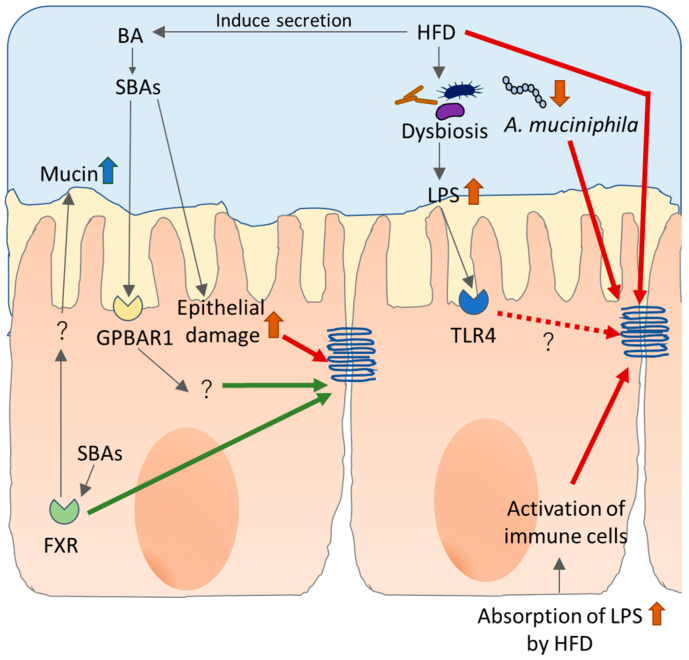
Effects of high-fat diet (HFD) and bile acids (BAs) on tight junction (TJ). Fatty acids impair TJ function directly and open the leak pathway. In addition, HFD affects the pollution of bacteria and exacerbates LPS production. Dysfunction of TJ lets LPS invade into epithelial cells where it becomes the target of immune cells, which provokes further damage to TJ. HFD also induces secretion of BAs which are converted to cytotoxic secondary BAs (SBAs) by bacteria. Nevertheless, SBAs show a protective effect on TJ function via G protein-coupled bile acid receptor (GPBAR1) and farnesoid X receptor (FXR).

Dietary fats-derived fatty acids are broadly categorized into saturated or unsaturated fatty acids. In parallel, there is another classification based on the length of fatty acids: SFCAs, middle-chain fatty acids (MCFAs), and long-chain fatty acid (LCFAs). They have the ability to affect paracellular permeability as demonstrated in previous in vitro studies [103,104]. Eicosapentaenoic acid (EPA), docosahexaenoic acid (DHA), and γ-linolenic acid, which belong to unsaturated LCFAs increase TJ permeability under normal physiological state without any disruptive stimuli to the intestinal barrier in Caco-2 cells [103,104]. However, these LCFAs decrease the TJ permeability in T84 cells, a cell line similar to Caco-2 cells. In addition, EPA and DHA reduce IL-4-mediated increase in paracellular permeability in T84 cells [105]. Both capric acid and lauric acid, known as saturated MCFAs, enhance the increase in paracellular permeability through activation of MLCK in Caco-2 cells [106]. Additionally, capric acid induces conformational alteration of TJ proteins including occludin and ZO-1 [107], whereas lauric acid does not [106]. These results indicate that dietary fats can alter intestinal permeability through the TJ-associated paracellular route by interacting with epithelial cells directly (Figure 5).

Etiological studies show the association of LCFAs with IBD progression, which implies that LCFAs alter the intestinal barrier function in humans. A large prospective cohort study demonstrated a relationship between higher consumption of DHA and a decrease in the incidence of ulcerative colitis (UC) [108]. Association of n-6 and n-3 polyunsaturated fatty acids (PUFAs) with IBD state has also been reported. Generally, n-6 PUFAs are suggested to trigger or enhance inflammatory signaling pathways, whereas n-3 PUFAs exhibit anti-inflammatory effects [109,110,111]. Higher intake of n-3 PUFA is associated with an increase in n-3/n-6 ratio in the erythrocyte membrane of the IBD patients who are in a remission state compared to those who had relapsed [112]. Serum n-3 fatty acids and EPA levels have been reported to be positively correlated with pro-inflammatory cytokine levels and disease activity, whereas serum n-6 fatty acids are inversely correlated with these indexes [113].

HFD can induce an increase in intestinal permeability in mice or rats with decreased mRNA or protein expression of TJ including claudins-1, claudins-2, claudins-3, and ZO-1 [114,115,116,117]. More specifically, in IL-10 knockout mice with IBD, IBD-like colitis is spontaneously triggered with an increase in intestinal permeability [118]. A high saturated fat diet was observed to promote Th1 immune response and increase the incidence of colitis [119]. These effects possibly arise due to an increased population of *Bilophila wadsworthi*, a sulfite-reducing pathobiont, through taurine-conjugation of hepatic bile acids. Mice fed a high-fat and high-sugar combination diet showed increased fecal inflammatory markers with increased levels of proteobacteria in the stool [120]. In accordance with these changes, these mice are more susceptible to DSS-induced colitis. HFD also affects IgA secretion. The level of secretory IgA coating the gut microbiota is elevated both in normal diet and HFD fed mice. However, this increase is diminished in HFD fed mice [121]. Hence, HFD diet induces an increase in intestinal permeability via downregulation of TJ and alters the immune response mediated by T cells and IgA-producing plasma cells in vivo.

#### 5.1.2. HFD-Induced LPS Absorption and Intestinal Barrier

Increased LPS absorption from leaky gut has been suggested by several studies on HFD-fed mice showing increased permeability with elevated serum LPS levels [114,122]. On the other hand, mice injected with LPS exhibited a reduction in plasma HDL cholesterol and elevations in plasma triglycerides [123]. A human cohort study also demonstrated that high serum LPS level in type 1 diabetic patients is associated with high serum triglyceride levels and increased diastolic blood pressure [124]. These results suggest that leaky gut affects dyslipidemia via absorption of LPS (Figure 5). In fact, mouse models have shown the importance of TLR4, a receptor for LPS, and its signaling in diet-induced insulin resistance and atherosclerosis [125]. TLR4 knockout mice are resistant to HFD-induced glucose intolerance [126,127] as well as HFD-induced atherosclerosis [128]. Similarly, human studies have shown that high fat and high carbohydrate intake modulates TLR2 and TLR4 expression in mononuclear cells with increased LPS level in the serum [129,130]. Besides, LPS stimulates T helper cell (Th) 17 differentiation though TLR4 in response to LPS [131] that may contribute to an LPS-induced inflammatory reaction, since IL-17A decreases the expression of peroxisome proliferator-activated receptor-α, which is anti-inflammatory and inhibits fatty-acid oxidation [132]. An advanced study has shown that leukocyte infiltration to the liver and mRNA expression of inflammatory cytokines TLR4 and TLR9 were increased in HFD-fed mice, in which the intestinal barrier was disrupted by co-administration of DSS with HFD, which downregulated ZO-1 and Claudin-1 expression in the colon, suggesting that leaky gut and the following attack to liver by LPS are involved in pathogenesis of liver dysfunction [133].

### 5.2. Alteration of Microbiome by HFD and Intestinal Permeability

Several studies have shown alteration of the microbiome by HFD both in animals [134,135,136] and humans [137,138]. A recent study demonstrated the possibility that HFD-induced alteration of the microbiome indirectly modulates the expression of TJ. Constant light exposure with HFD induces glucose abnormalities, insulin resistance, inflammation, and liver steatohepatitis in mice, which is associated with less abundance of *Butyricicoccus*, *Clostridium*, and *Turicibacter*, also known as butyrate producers. These changes correlate with decreased butyrate levels in colon contests, decreased colon expressions of occludin-1 and ZO-1, and increased serum LPS and mRNA expression of liver LPS-binding protein [139]. As described earlier, *A. muciniphila* exhibits protective effects on intestinal permeability. The effect of these bacteria on intestinal barrier dysfunction is induced by HFD and associated systemic disorders, including obesity and T2D. An abundance of *A. muciniphila* is negatively correlated with fasting blood glucose, waist-to-hip ratio, and subcutaneous fat cell diameter in obese subjects and an increased abundance of *A. muciniphila* positively correlates with improvement in insulin sensitivity markers and other clinical parameters after calorie restriction [140]. Compared to healthy subjects, T2D patients harbor less fecal *A. muciniphila* extracellular vehicles (AmEVs) [141], which suppresses HFD-induced weight gain, increased plasma cholesterol, triglycerides, and glucose, along with downregulation of intestinal TLR4 and TJ mRNAs in mice [142]. In addition, the membrane protein derived from *A. muciniphila* reduces intolerance to glucose and plasma LPS levels along with upregulation of the molecules related to insulin signaling and claudin-3 [143]. These results imply that *A. muciniphila* exhibits a beneficial effect on HFD-induced disorders and intestinal barrier integrity might be involved in this mechanism (Figure 5).

### 5.3. Bile Acids and Intestinal Permeability

Bile acids (BAs) secreted from duodenum help in digestion of lipids as well as cholesterol and fat-soluble vitamins. HFD induce enhanced BA discharge, resulting in increased colonic concentrations of primary BAs, compared with low or normal fat diets [144]. However, 5% to 10% of BAs are not reabsorbed but are converted to secondary bile acids by bacteria in the large intestine. Secondary bile acids are harmful and are suggested to promote colon carcinogenesis [145]. In addition to their role in dietary lipid absorption and cholesterol homeostasis, bile acids act as signaling molecules via two major signaling pathways. One is G protein-coupled bile acid receptor (GPBAR1, also known as TGR5) and another comprises members of the nuclear hormone receptor superfamily including the farnesoid X receptor (FXR) [146]. Animal studies indicate that both GPBAR1 and FXR contribute to the integrity of the intestinal barrier. GPBAR1 knock out mice show abnormal molecular architecture of epithelial TJ with increased expression and abnormal subcellular distribution of zonulin-1 with increased intestinal permeability. In addition, these mice are more sensitive to DSS stimuli and exhibit severe colitis [147]. Activation of GPBAR1 with synthetic agonist reversed intestinal inflammation in chemically induced colitis models. The possible mechanisms include reduction in the trafficking of monocytes from blood to intestinal mucosa and modulation of the activation state of macrophages which results in decreased expression of inflammatory genes, including TNF-a, IFN-g, IL-1b, IL-6, and CCL2 [148]. Intestinal permeability is increased in FXR knockout mice [149] and FXR-agonist exhibits a protective effect on chemically induced colitis with reduced epithelial permeability and several inflammatory responses [150]. A recent study demonstrated that gut-specific FXR-deficient mice exhibit increased intestinal permeability, possibly due to reduced mucosal integrity, associated with decreased secretion of mucin 2 protein and lower levels of E-cadherin protein [151]. Taken together, these results indicate that BAs maintain the intestinal barrier through the BA sensing receptor at least partially (Figure 5).

## 6. Concluding Remarks

We reviewed the effect of dietary fiber and fats on intestinal permeability and microbiome, and the correlations between them. Dietary fiber is recognized as a protective nutrient for the intestinal barrier and contributes to maintaining the microbiota in a healthy state. These beneficial effects are mediated by SCFAs, and epithelial IL-18 seems to be involved in a molecular mechanism of barrier-regulation. In contrast, fats likely promote an increase in epithelial permeability. In vitro studies show that fatty acids directly impair the epithelial barrier. Besides, fat-induced increased plasma LPS levels are able to disrupt the intestinal barrier. These findings indicate that a westernized diet contains high fats and poor fibers likely to induce or exacerbate various systemic diseases due to bad intestinal health including impaired intestinal barrier function. In addition, both dietary fiber and high fat can affect the relative population of *A. muciniphila* which imparts a protective effect on intestinal barrier integrity and disease state, suggesting possible causative relationships between dietary fibers and fats, microbiome, LGS, and systemic diseases. Nevertheless, these relationships have a missing link since the molecules which are responsible for LGS and the successive dysfunction of various organs are not identified. To clarify this point, we should identify the precise region of LGS which conventional LGS-evaluating reagents cannot do [45]. This is the bottleneck to evaluate whether the subjects are LGS or not in animals and clinical studies. That is why leaky gut is not something that can be concluded as easily so far, especially in humans. Improvement of LGS-evaluating methods or development of an ex vivo organ like an organoid would connect the missing link and provide more information about the relationship between nutrients and LGS.
